# Supplementary material for: The Regulatory Hub of Siderophore Biosynthesis in the Phytopathogenic Fungus Alternaria alternata
Source: J Fungi (Basel). 2023 Mar 29;9(4):427. doi: 10.3390/jof9040427 (PMC10146468; doi:10.3390/jof9040427)
Supplement: Supplementary file 1 [file jof-09-00427-s001.zip › Supplementary_Fig._S1_S13.pdf]

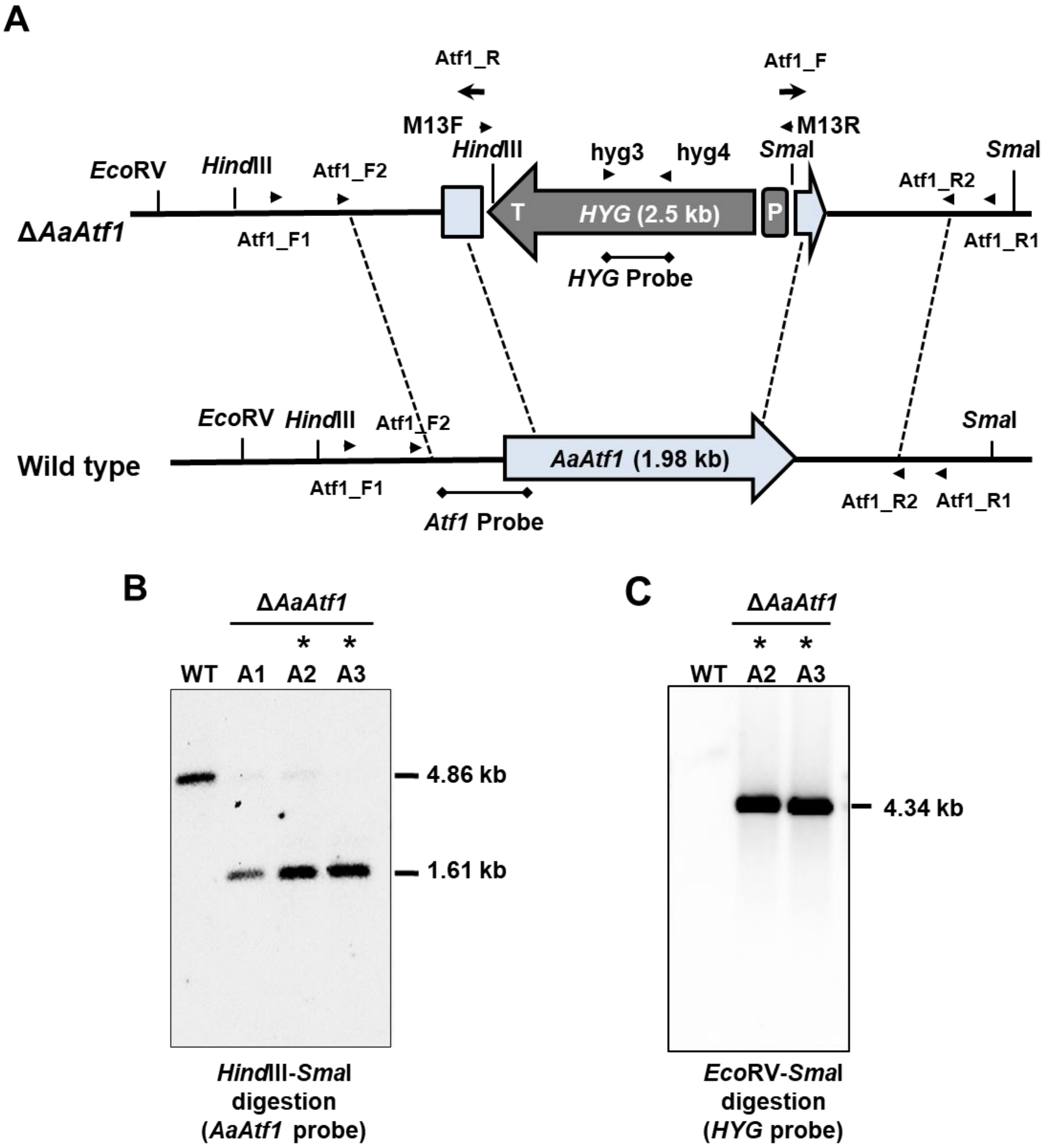


**Figure S1. Deletion of the *AaAtf1* gene using a split marker approach in the tangerine pathotype of *A. alternata*.** (**A**) Schematic depiction of gene disruption of *AaAtf1* via homologous recombination using truncated but overlapping DNA fragments of a hygromycin phosphotransferase-coding gene (*HYG*) under control by the *Aspergillus nidulans trpC* promoter (P) and terminator (T). Split marker fragments were amplified with the primers as indicated. (**B**)(**C**) Southern-blot analysis of genomic DNA prepared from wild-type (WT), three putative *AaAtf1* mutants (A1, A2, and A3). Fungal DNA was cleaved with *Hind*III and *Sma*I (**B**), or *Eco*RV and *Sma*I (**C**), electrophoresed, blotted to a nylon membrane and hybridized with an *AaAtf1* (**B**) or a *HYG* (**C**) probe. The Δ*AaAtf1* mutants (A2 and A3) used in this research are indicated by asterisks.

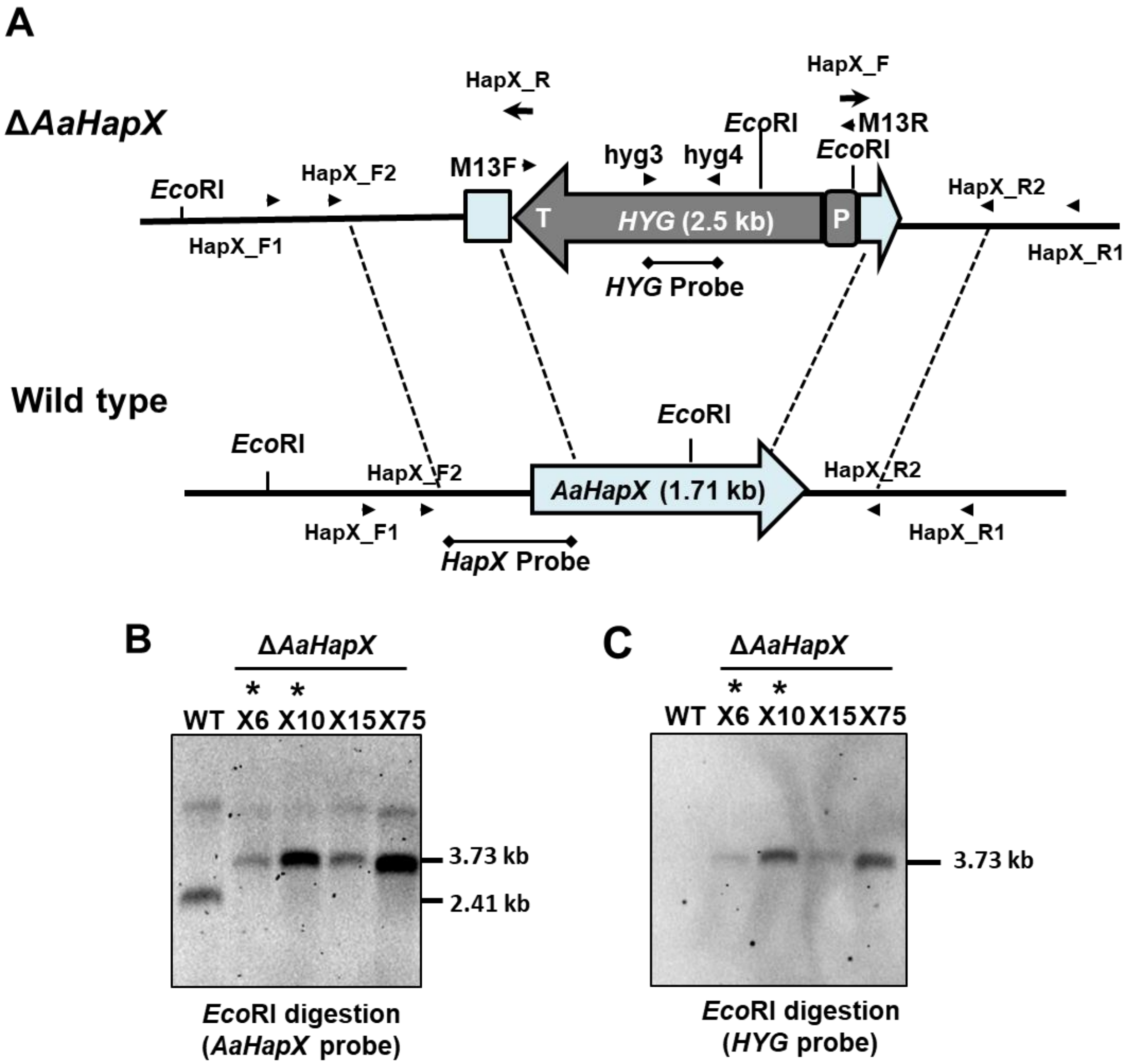


**Figure S2. Deletion of the *AaHapX* gene using a split marker approach in the tangerine pathotype of *A. alternata*.** (**A**) Schematic depiction of gene disruption of *AaHapX* via homologous recombination using truncated but overlapping DNA fragments of a hygromycin phosphotransferase-coding gene (*HYG*) under control by the *Aspergillus nidulans trpC* promoter (P) and terminator (T). Split marker fragments were amplified with the primers as indicated. (**B**)(**C**) Southern-blot analysis of genomic DNA prepared from wild-type (WT) and four putative Δ*AaHapX* mutants (X6, X10, X15, and X75). Fungal DNA was cleaved with *Eco*R1, electrophoresed, blotted to a nylon membrane, and hybridized with an *AaHapX* (**B**) or a *HYG* (**C**) probe. The Δ*AaHapX* mutants (X6 and X10) used in this research are indicated by asterisks.

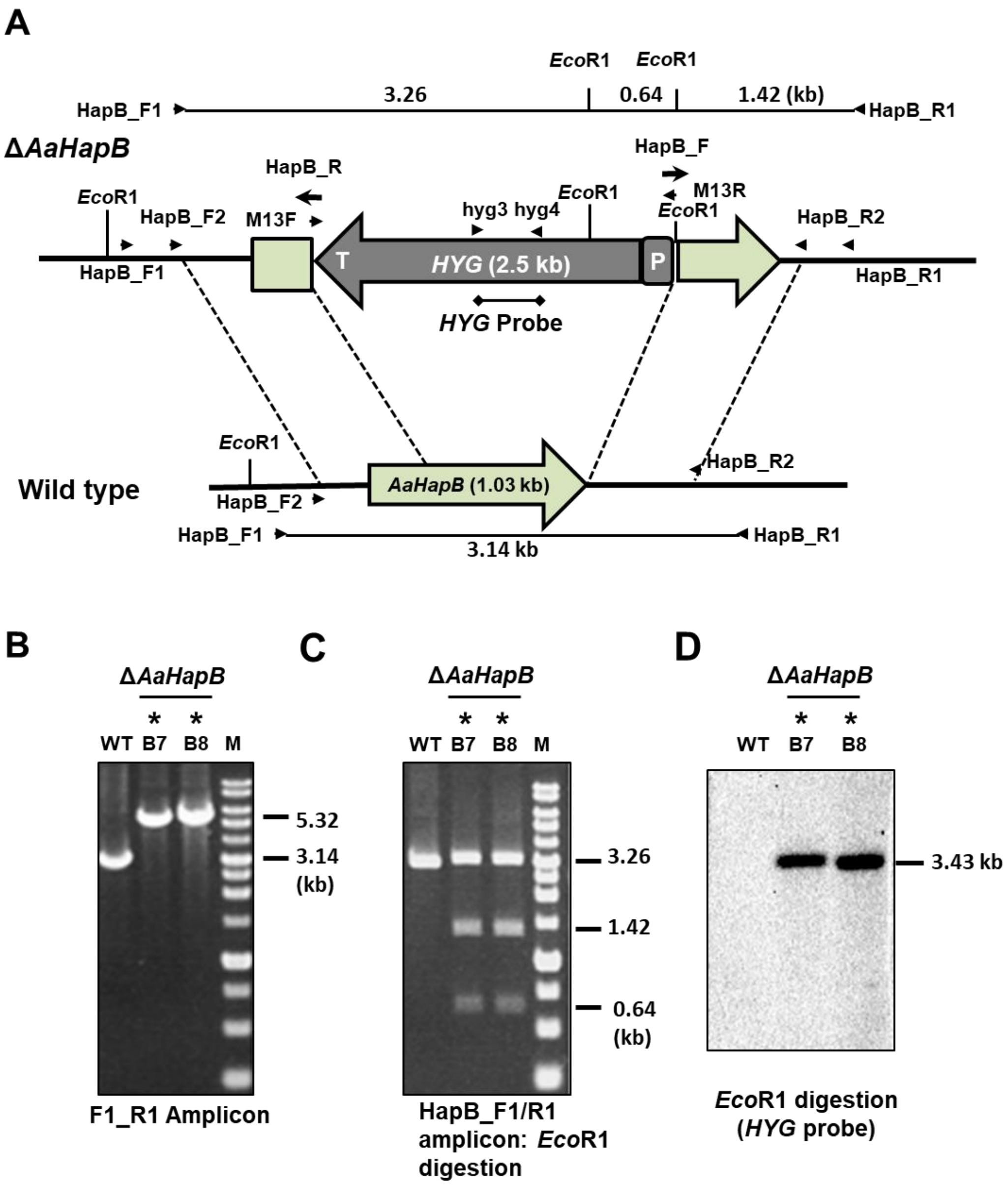


**Figure S3. Deletion of the *AaHapB* gene using a split marker approach in the tangerine pathotype of *A. alternata*.** (**A**) Schematic depiction of gene disruption of *AaHapB* via homologous recombination using truncated but overlapping DNA fragments of a hygromycin phosphotransferase-coding gene (*HYG*) under control by the *Aspergillus nidulans trpC* promoter (P) and terminator (T). Split marker fragments were amplified with the primers as indicated. (**B**) Gel image showing DNA fragments amplified from wild-type (WT) and two putative Δ*AaHapB* mutants (B7 and B8) with the primers HapB_F1/HapB_R1. (**C**) Restriction enzyme length polymorphism patterns of the F1/R1 amplicons after *Eco*R1 digestion. (**D**) Southern-blot analysis of genomic DNA prepared from wild-type (WT) and two transformants (B7 and B8). Fungal DNA was cleaved with *Eco*RI, electrophoresed, blotted to a nylon membrane, and hybridized with a *HYG* probe. The Δ*AaHapB* mutants (B7 and B8) used in this research are indicated by asterisks.

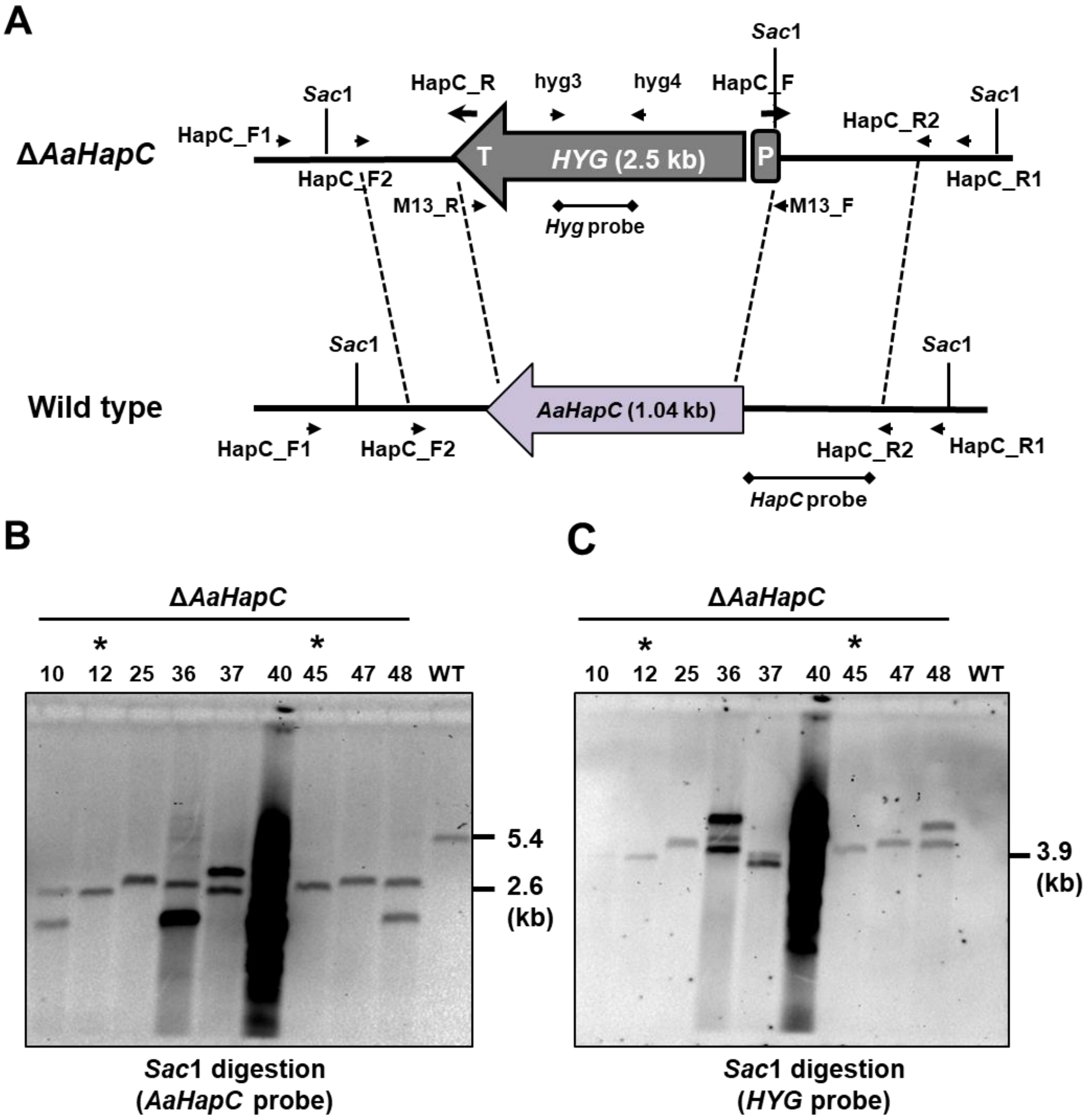


**Figure S4. Deletion of the *AaHapC* gene using a split marker approach in the tangerine pathotype of *A. alternata*.** (**A**) Schematic depiction of gene disruption of *AaHapC* via homologous recombination using truncated but overlapping DNA fragments of a hygromycin phosphotransferase-coding gene (*HYG*) under control by the *Aspergillus nidulans trpC* promoter and terminator. Split marker fragments were amplified with the primers as indicated. (**B**)(**C**) Southern-blot analysis of genomic DNA prepared from wild-type (WT) and nine transformants (10, 12, 25, 36, 37, 40, 45, 47, and 48). Fungal DNA was cleaved with *Sac*I, electrophoresed, blotted to a nylon membrane, and hybridized with an *AaHapB* (**B**) or a *HYG* (**C**) probe. The Δ*AaHapC* mutants (C12 and C45) used in this research are indicated by asterisks.

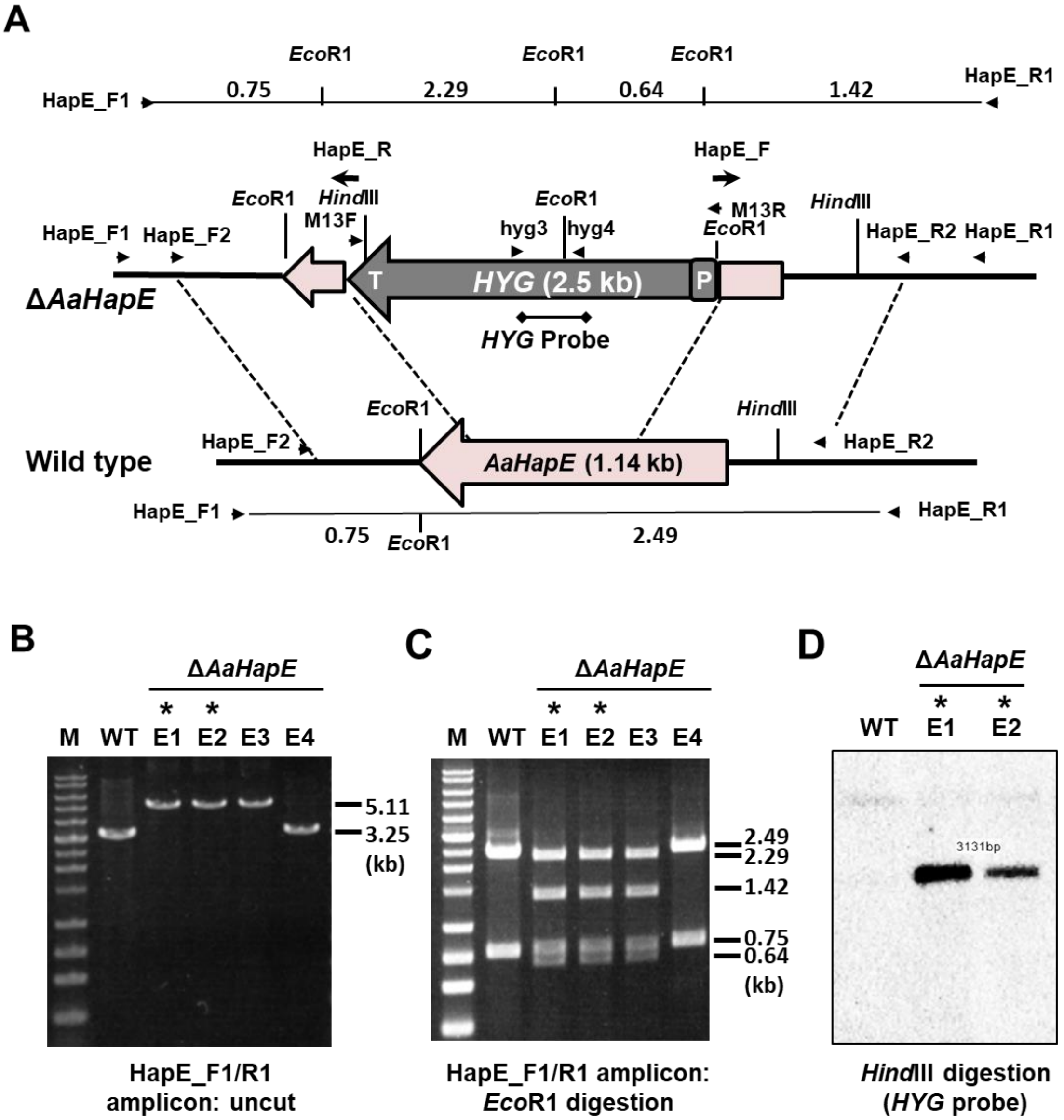


**Figure S5. Deletion of the *AaHapE* gene using a split marker approach in the tangerine pathotype of *A. alternata*.** (**A**) Schematic depiction of gene disruption of *AaHapE* via homologous recombination using truncated but overlapping DNA fragments of a hygromycin phosphotransferase-coding gene (*HYG*) under control by the *Aspergillus nidulans trpC* promoter (P) and terminator (T). Split marker fragments were amplified with the primers as indicated. (**B**) Gel image showing DNA fragments amplified from wild-type (WT), four transformants (E1, E2, E3, and E4) with the primers HapE_F1/HapE_R1. (**C**) Restriction enzyme length polymorphism patterns of the F1/R1 amplicons after *Eco*R1 digestion. (**D**) Southern-blot analysis of genomic DNA prepared from wild-type (WT) and two transformants (E1 and E2). Fungal DNA was cleaved with *Hind*III, electrophoresed, blotted to a nylon membrane, and hybridized with a *HYG* probe. The Δ*AaHapE* mutants (E1 and E2) used in this research are indicated by asterisks.

# Atf1

Distance

| | | 1 | 2 | 3 | 4 | 5 | 6 | 7 | 8 |
|---|---|---|---|---|---|---|---|---|---|
| *Aspergillus fumigatus* Af293 | 1 | | 0.21 | 0.31 | 0.61 | 0.61 | 0.70 | 0.66 | 0.64 |
| *Aspergillus niger* ATCC 1015 | 2 | 73.94 | | 0.30 | 0.65 | 0.65 | 0.71 | 0.65 | 0.65 |
| *Penicillium digitatum* PHI26 | 3 | 62.81 | 65.74 | | 0.59 | 0.60 | 0.61 | 0.66 | 0.64 |
| *Fusarium oxysporum* f. sp. *lycopersici* 4287 | 4 | 43.31 | 44.54 | 44.17 | | 0.07 | 0.36 | 0.70 | 0.68 |
| *Fusarium graminearum* PH-1 | 5 | 43.23 | 44.64 | 44.25 | 92.61 | | 0.39 | 0.69 | 0.67 |
| *Magnaporthe oryzae* 70-15 | 6 | 39.60 | 41.68 | 43.72 | 64.05 | 62.52 | | 0.78 | 0.77 |
| *Alternaria alternata* EV-MIL-31 | 7 | 40.13 | 41.71 | 39.80 | 41.02 | 41.46 | 37.42 | | 0.00 |
| *Alternaria alternata* SRC1lrK2f | 8 | 40.87 | 43.22 | 40.41 | 41.81 | 42.25 | 38.40 | 90.81 | |

Identity (%)

**Conserved Motif**

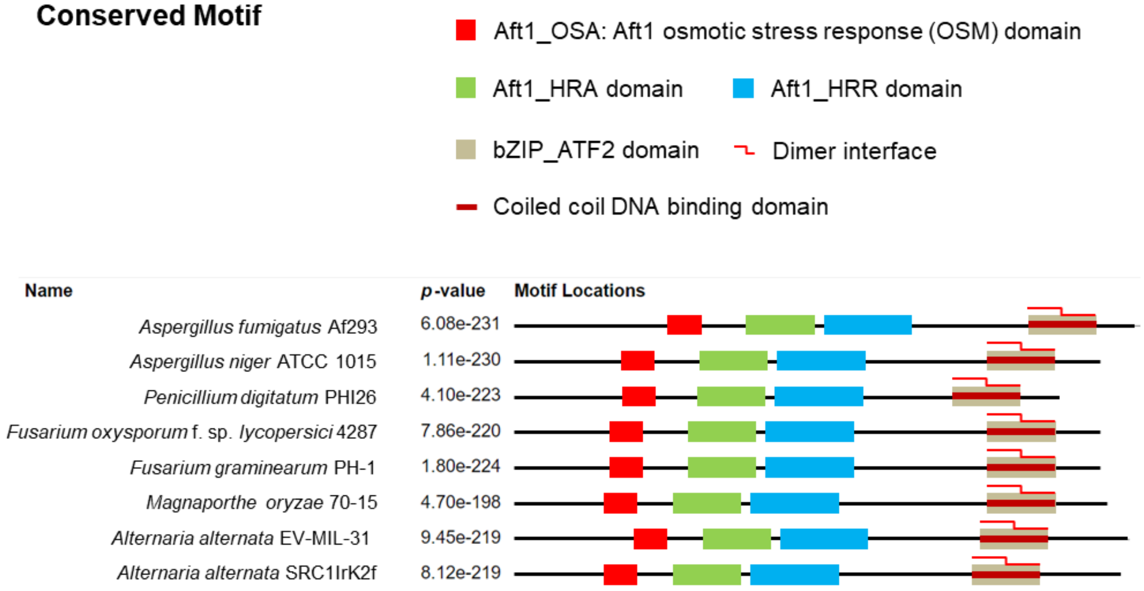


**Figure S6. Pairwise comparison and alignment of conserved motifs of the AaAtf1 sequence with fungal Atf1 orthologs.** A pairwise comparison of the sequence was performed using CLC Genomic Workbench 9.5.1 to calculate genetic distance using the jukes-cantor model and percent identity. Conserved domains were predicted by NCBI and the MEME Suite server.

# HapX

Distance

| | | 1 | 2 | 3 | 4 | 5 | 6 | 7 | 8 |
|---|---|---|---|---|---|---|---|---|---|
| *Aspergillus fumigatus* Af293 | 1 | | 0.21 | 0.35 | 0.88 | 0.84 | 0.86 | 1.26 | 1.26 |
| *Aspergillus niger* ATCC 1015 | 2 | 76.85 | | 0.33 | 0.86 | 0.80 | 0.89 | 1.20 | 1.20 |
| *Penicillium digitatum* PHI26 | 3 | 65.00 | 65.70 | | 0.90 | 0.86 | 0.91 | 1.32 | 1.32 |
| *Fusarium oxysporum* f. sp. *lycopersici* 4287 | 4 | 24.41 | 25.28 | 24.61 | | 0.13 | 0.68 | 1.22 | 1.22 |
| *Fusarium graminearum* PH-1 | 5 | 30.97 | 32.25 | 31.38 | 72.06 | | 0.68 | 1.23 | 1.23 |
| *Magnaporthe oryzae* 70-15 | 6 | 29.10 | 28.23 | 28.76 | 37.99 | 45.26 | | 1.34 | 1.35 |
| *Alternaria alternata* EV-MIL-31 | 7 | 17.73 | 18.38 | 17.55 | 15.96 | 19.43 | 17.52 | | 0.00 |
| *Alternaria alternata* SRC1lrK2f | 8 | 17.73 | 18.38 | 17.55 | 15.96 | 19.43 | 17.39 | 99.81 | |

Identity (%)

**Conserved Motif**

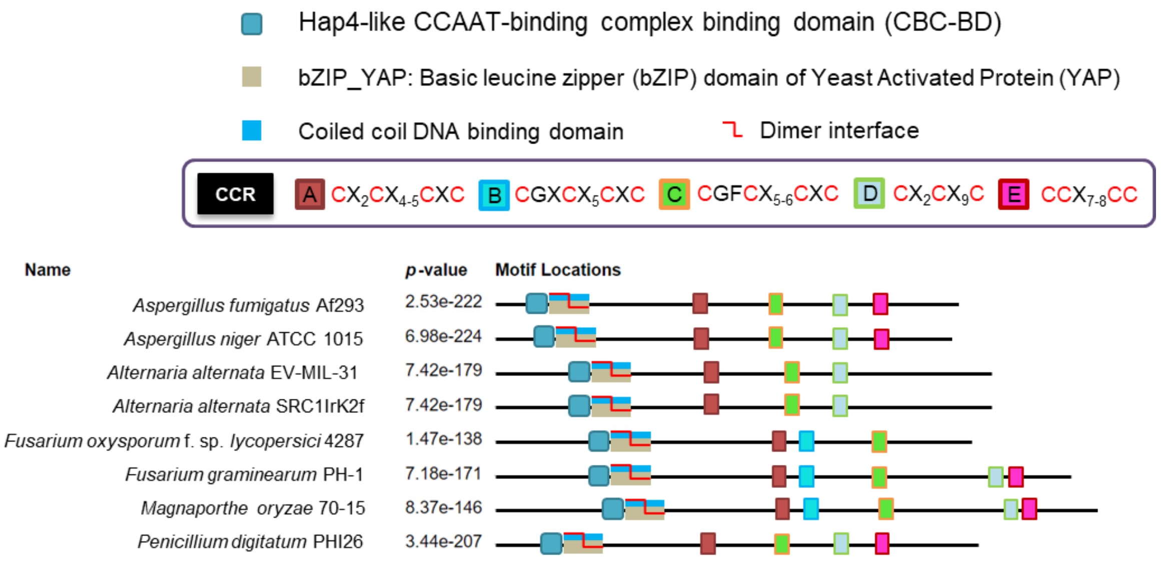


**Figure S7. Pairwise comparison and alignment of conserved motifs of the AaHapX sequence with fungal HapX orthologs.** A pairwise comparison of the sequence was performed using CLC Genomic Workbench 9.5.1 to calculate genetic distance using the jukes-cantor model and percent identity. Conserved domains containing cysteine (C) residues were predicted by NCBI and the MEME Suite server.

# HapB

Distance

| | | 1 | 2 | 3 | 4 | 5 | 6 | 7 | 8 |
|---|---|---|---|---|---|---|---|---|---|
| *Aspergillus fumigatus* Af293 | 1 | | 0.12 | 0.31 | 0.68 | 0.68 | 0.62 | 0.63 | 0.67 |
| *Aspergillus niger* ATCC 1015 | 2 | 68.02 | | 0.25 | 0.60 | 0.60 | 0.53 | 0.53 | 0.57 |
| *Penicillium digitatum* PHI26 | 3 | 71.62 | 59.73 | | 0.69 | 0.69 | 0.63 | 0.63 | 0.70 |
| *Alternaria alternata* EV-MIL-31 | 4 | 44.62 | 36.41 | 43.92 | | 0.00 | 0.65 | 0.71 | 0.72 |
| *Alternaria alternata* SRC1lrK2f | 5 | 44.62 | 36.41 | 43.92 | 100.00 | | 0.65 | 0.71 | 0.72 |
| *Fusarium oxysporum* f. sp. *lycopersici* 4287 | 6 | 41.58 | 36.74 | 41.33 | 39.67 | 39.67 | | 0.13 | 0.41 |
| *Fusarium graminearum* PH-1 | 7 | 41.84 | 37.19 | 42.13 | 38.32 | 38.32 | 86.64 | | 0.38 |
| *Magnaporthe oryzae* 70-15 | 8 | 41.22 | 35.34 | 40.16 | 38.74 | 38.74 | 61.71 | 63.01 | |

Identity (%)

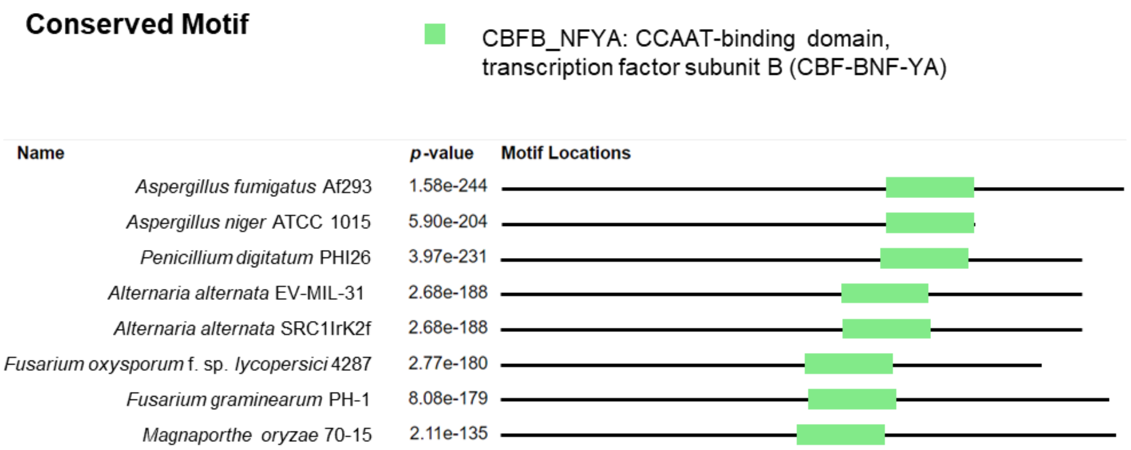


**Figure S8. Pairwise comparison and alignment of conserved motifs of the AaHapB sequence with fungal HapB orthologs.** A pairwise comparison of the sequence was performed using CLC Genomic Workbench 9.5.1 to calculate genetic distance using the jukes-cantor model and percent identity. Conserved domains were predicted by NCBI and the MEME Suite server.

# HapC

Distance

| | | 1 | 2 | 3 | 4 | 5 | 6 | 7 | 8 |
|---|---|---|---|---|---|---|---|---|---|
| *Aspergillus fumigatus* Af293 | 1 | | 0.16 | 0.26 | 0.54 | 0.47 | 0.44 | 0.45 | 0.38 |
| *Aspergillus niger* ATCC 1015 | 2 | 79.82 | | 0.26 | 0.50 | 0.44 | 0.45 | 0.46 | 0.39 |
| *Penicillium digitatum* PHI26 | 3 | 61.00 | 63.75 | | 0.53 | 0.48 | 0.48 | 0.50 | 0.42 |
| *Fusarium oxysporum* f. sp. *lycopersici* 4287 | 4 | 39.93 | 40.82 | 35.03 | | 0.14 | 0.35 | 0.46 | 0.41 |
| *Fusarium graminearum* PH-1 | 5 | 51.06 | 56.00 | 48.00 | 63.71 | | 0.34 | 0.39 | 0.34 |
| *Magnaporthe oryzae* 70-15 | 6 | 53.22 | 56.50 | 48.59 | 49.60 | 67.31 | | 0.41 | 0.34 |
| *Alternaria alternata* EV-MIL-31 | 7 | 50.22 | 51.79 | 44.94 | 38.76 | 54.63 | 53.00 | | 0.12 |
| *Alternaria alternata* SRC1lrK2f | 8 | 44.54 | 46.40 | 39.68 | 35.89 | 49.29 | 49.06 | 72.87 | |

Identity (%)

**Conserved Motif**

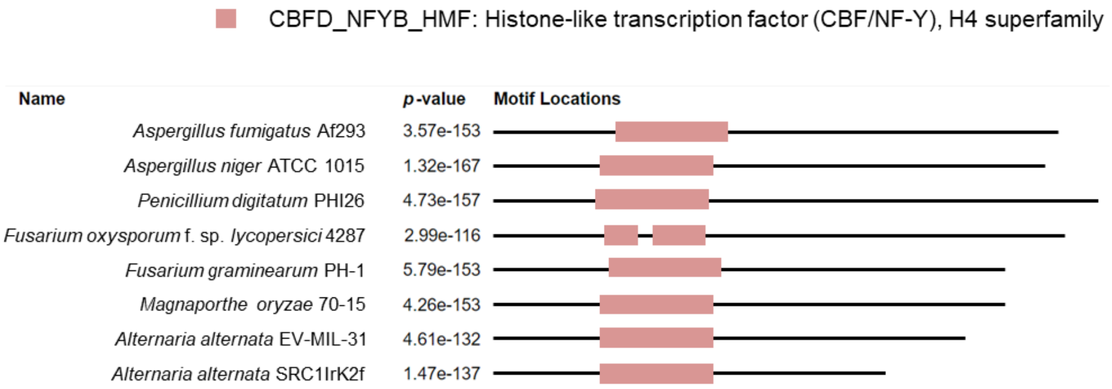


**Figure S9. Pairwise comparison and alignment of conserved motifs of the AaHapC sequence with fungal HapC orthologs.** A pairwise comparison of the sequence was performed using CLC Genomic Workbench 9.5.1 to calculate genetic distance using the jukes-cantor model and percent identity. Conserved domains were predicted by NCBI and the MEME Suite server.

# HapE

Distance

| | | 1 | 2 | 3 | 4 | 5 | 6 | 7 |
|---|---|---|---|---|---|---|---|---|
| *Fusarium oxysporum* f. sp. *lycopersici* 4287 | 1 | | 0.21 | 0.45 | 0.47 | 0.43 | 0.60 | 0.60 |
| *Fusarium graminearum* PH-1 | 2 | 79.14 | | 0.37 | 0.44 | 0.41 | 0.54 | 0.54 |
| *Magnaporthe oryzae* 70-15 | 3 | 57.89 | 63.29 | | 0.46 | 0.49 | 0.54 | 0.54 |
| *Aspergillus fumigatus* Af293 | 4 | 56.10 | 57.04 | 57.39 | | 0.09 | 0.48 | 0.48 |
| *Aspergillus niger* ATCC 1015 | 5 | 57.34 | 57.59 | 56.27 | 86.50 | | 0.46 | 0.46 |
| *Alternaria alternata* EV-MIL-31 | 6 | 45.79 | 49.22 | 47.98 | 50.46 | 50.62 | | 0.00 |
| *Alternaria alternata* SRC1lrK2f | 7 | 45.79 | 49.22 | 47.98 | 50.46 | 50.62 | 100.00 | |

Identity (%)

### Conserved Motif

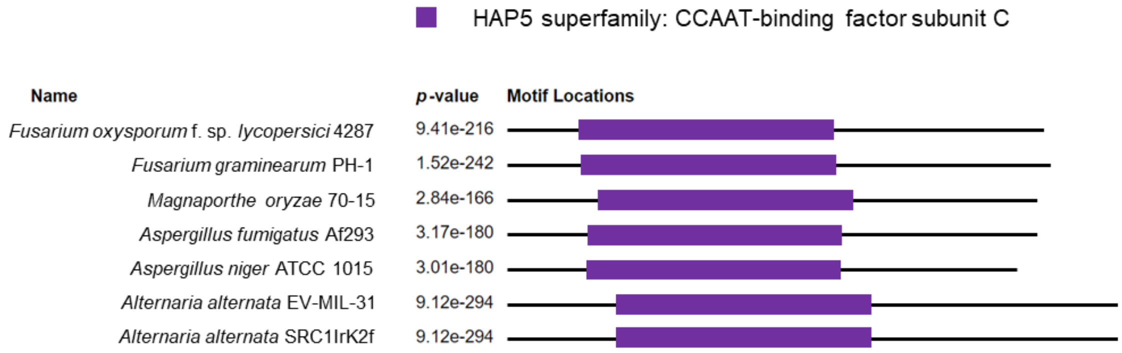


**Figure S10. Pairwise comparison and alignment of conserved motifs of the AaHapE sequence with fungal HapE orthologs.** A pairwise comparison of the sequence was performed using CLC Genomic Workbench 9.5.1 to calculate genetic distance using the jukes-cantor model and percent identity. Conserved domains were predicted by NCBI and the MEME Suite server.

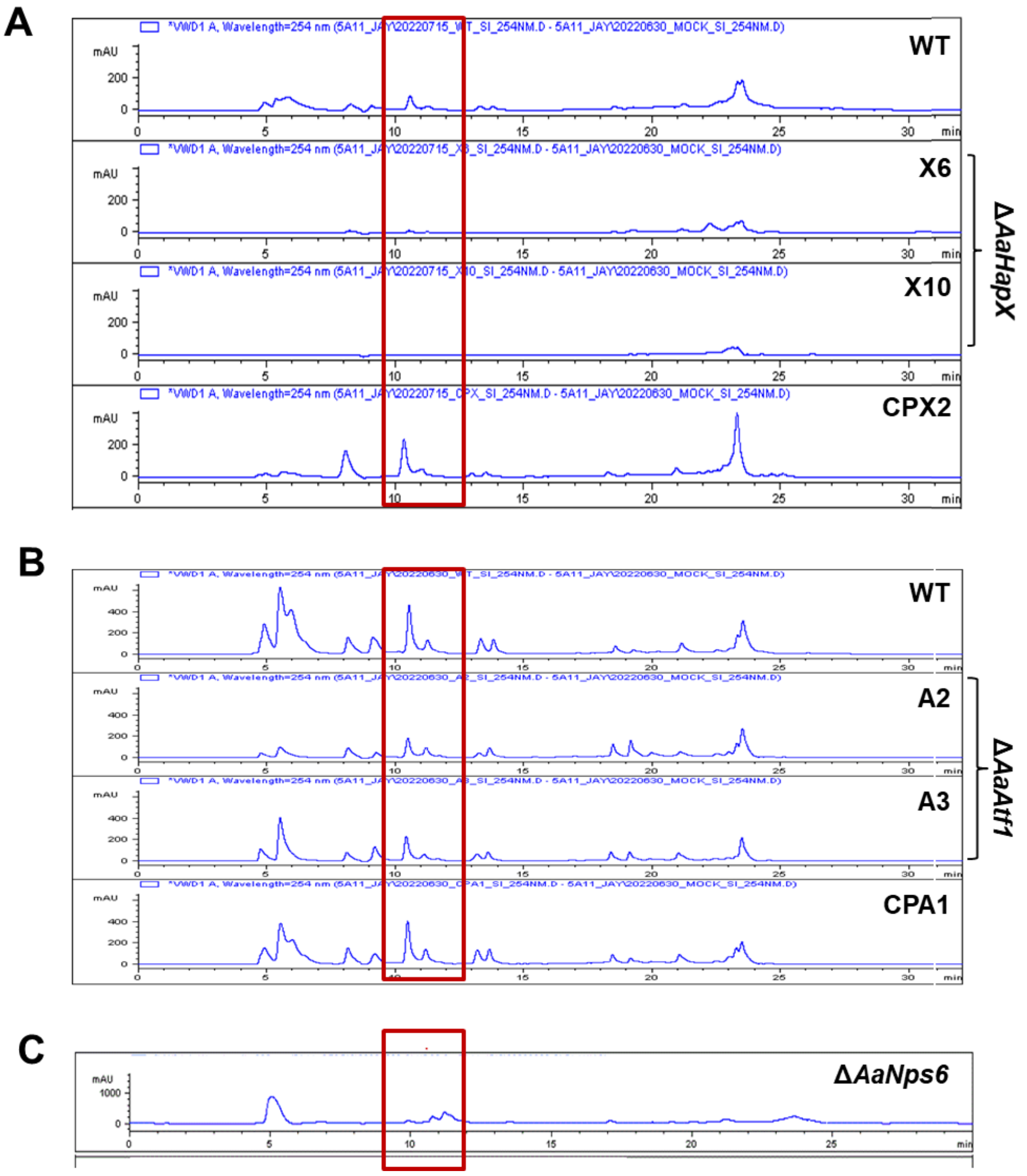


**Figure S11. HPLC analyses of siderophores isolated from the strains of *A. alternata*.** (**A**) WT: wild type, Δ*AaHapX*: the *AaHapX* deletion mutants (X6 and X10), and CPX2: the *AaHapX* complementation strain. (**B**) Δ*AaAtf1*: the *AaAtf1* deletion mutants (A2 and A3) and CPA1: the *AaAtf1* complementation strain. (**C**) Δ*AaNps6*: the *AaNps6* deletion mutant defective for a nonribosomal peptide synthetase involved in the biosynthesis of siderophores. Siderophores (indicated by red rectangle boxes) were separated in a reversed-phase Supelco Ascentis C-18 column (Sigma-Aldrich) using a mobile phase consisting of a linear gradient of 0.2% formic acid in water and 0.2% formic acid in methanol (from 75:25 to 55:45 in 10 min, 25:75 in 5 min, and 0:100 for final 5 min).

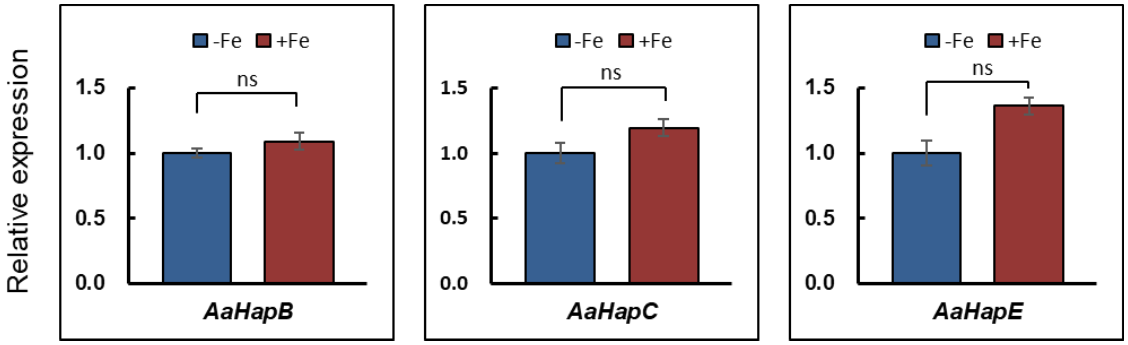


**Figure S12. qRT-PCR analyses.** No significant difference in the expression of *AaHapB*, *AaHapC*, and *AaHapE* in the wild type of *A. alternata* in response to iron. Fungal mycelium grown in PDB for 2 days was harvested, washed with sterile water, shifted to a minimal medium (MM) amended with or without 0.2 mM $FeCl_3$, and incubated for an additional 24 h. The relative expression level from three independent reactions was calculated by a comparative Ct method (*ΔΔCT*) in relation to the expression of the fungal β-tubulin-coding gene. In each assay, the expression level in the wild type grown in MM was set to 1, and subject to statistical analysis of Student’s t-test ($p < 0.01$). All experiments were repeated at least two times, showing similar trends.

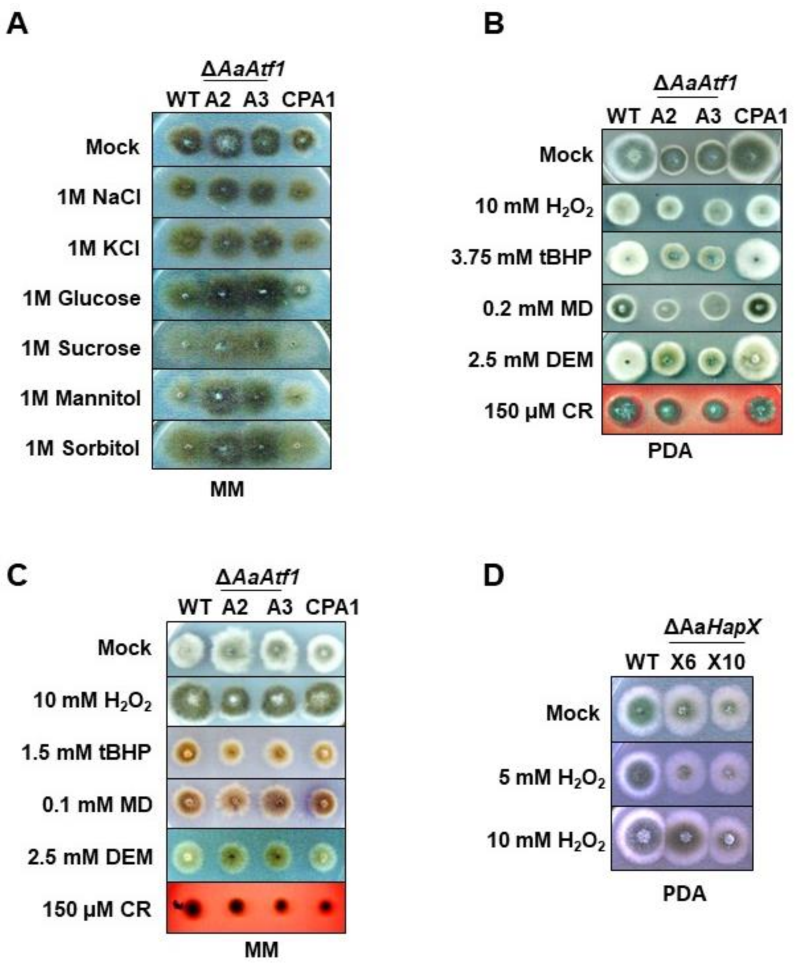


**Figure S13. Sensitivity tests.** Fungal strains were grown on MM or PDA amended with or without a chemical at 28°C for 3 to 7 days. Abbreviations: tBHP, *tert*-butyl-hydroperoxide; MD, menadione; DEM, diethyl maleate; and CR, Congo red.
