# Supplementary material for: The Regulatory Hub of Siderophore Biosynthesis in the Phytopathogenic Fungus Alternaria alternata
Source: J Fungi (Basel). 2023 Mar 29;9(4):427. doi: 10.3390/jof9040427 (PMC10146468; doi:10.3390/jof9040427)
Supplement: Supplementary file 1 [file jof-09-00427-s001.zip › Supplementary_Table_S1.pdf]

Table S1. Oligonucleotide primers

**Name**	**Sequence (5' -> 3')**	Note
HYG		
M13F	GCCAGGGTTTTCCCAGTCACGAC	
M13R	GCGGATAACAATTTCACACAGGA	
hyg3	GGATGCCTCCGCTCGAAGTA	
hyg4	CGTTGCAAGACCTGCCTGAA	
HapX		
HapX_F1	GATCAACGCAAATCAACGCTCGA	
HapX_F2	CCAGACGTTTTCTCTCTCACGCTA	
HapX_R	GTCGTGACTGGGAAAACCCTGGCGTGTTGCTGGTGGCAGACAAC	The underlined region is complementary to M13F sequence
HapX_F	TCCTGTGTGAAATTGTTATCCGCGCATGTACAGTGCGATGGAGCTA	The underlined region is complementary to M13R sequence
HapX_R2	CGGCATGGTGCTACATTGTTCT	
HapX_R1	CGTTGGTAGCTCAAGATCGAGTTGA	
Comp_HapX_F	aaa*GGATCC* TACTCGGAGCAAGCACTTG	The underlined region is the attached *Bam* HI cutting site
Comp_HapX_R	ccc*AAGCTT* ATGGTCAATGGACTGGAC	The underlined region is the attached *Hind* III cutting site
HapB		
HapB_F1	GCAATGACCAATCCACTACTGTGGA	
HapB_F2	CACGATCCTCAGCAGTGGGAA	
HapB_R	CTGGGAAAACCCTGGCAAAACCCTGGCGTTCATACCTGCATGAGTCCAGACAC	The underlined region is complementary to M13F sequence
HapB_F	TCCTGTGTGAAATTGTTATCCGCGACACCGACATACGTGGAGAT	The underlined region is complementary to M13R sequence
HapB_R2	CAACACACAGCCCACCAAGC	
HapB_R1	CCACAGACCGTGGAGCAATAGA	
Comp_HapB_F	ccc*GTCGAC* ACCAATCCACTACTGTGGAG	The underlined region is the attached *Sal* I cutting site
Comp_HapB_R	aaa*GCGGCCGC* TGCTGCAGACCACAACAC	The underlined region is the attached *Not* I cutting site
HapC		
HapC_F1	GCGGCCGCGCTTACAGAGCATCGGAAGCT	
HapC_F2	CTGCCAGATGTATTCCCAAG	
HapC_R	GTCGTGACTGGGAAAACCCTGGCGATGTTGAGGTCATATGTCC	The underlined region is complementary to M13F sequence
HapC_F	TCCTGTGTGAAATTGTTATCCGCCTGTTCTTCGTCAGGCGAC	The underlined region is complementary to M13R sequence
HapC_R2	CATACAGGATCGCCAGCTAG	
HapC_R1	GCTGAATGTCATCTGTGGCTGCA	
HapE		
HapE_F1	CGCTTCTAGTTTCGTGGGTGCT	
HapE_F2	GGACGTGATGTGATGTGGATCTT	
HapE_R	CTGGGAAAACCCTGGCAAAACCCTGGCGTCAATCGTCGGCAGCAGTCG	The underlined region is complementary to M13F sequence
HapE_F	TCCTGTGTGAAATTGTTATCCGCGTTGCCCTGCTGGCCATCAT	The underlined region is complementary to M13R sequence
HapE_R2	CATCTGAAGTGTGCTGCCCT	
HapE_R1	GACAGGGAAAAGCAGTGGGAA	
Comp_HapE_F	ccc*GTCGAC* TCTGTTGGACCCAGGAAG	The underlined region is the attached *Sal* I cutting site
Comp_HapE_R	aaa*GCGGCCGC* TGATTCCGTTGCGCTCAG	The underlined region is the attached *Not* I cutting site
Atf1		
Atf1_F1	TGGCTTTTGGTCGAGGGCCATC	
Atf1_F2	TGGCACCTAGCGGCAGACTTTGATTACG	
Atf1_R	GTCGTGACTGGGAAAACCCTGGCAAAACCCTGGCGTTCGTTTGGGTTGCGGCTGCCATA	The underlined region is complementary to M13F sequence
Atf1_F	TCCTGTGTGAAATTGTTATCCGCGACGGCCGGTATCCATCAAAGTAT	The underlined region is complementary to M13R sequence
Atf1_R2	GCCAGCCCCATGAATCCCTGCCGTT	
Atf1_R1	AACCAACCACTCCCTGCGCATG	
Comp_Atf1_F	gcg*GAATTC* AGGGGACCATGAGTACTCTTGGAG	The underlined region is the attached *Eco* RI cutting site
Comp_Atf1_R	gct*GGATCC* TCAGGAGGAGGGGGAGGAATTGC	The underlined region is the attached *BamH* I cutting site
Q-RT-PCR		
Tub_qF	TTCGTCGGTAACTCCACCTCCATC	*β-tubulin*
Tub_qR	ACTCAGCCTCAGTGAACTCCATCTC	
SreA_qF	CTGGTGGAGGGCGATGTA	*SreA*
SreA_qR	GAACGCTGGACAGCCACT	
HapX_qF	CGATATGGACTGGCGTCAAC	*HapX*
HapX_qR	GCTTGCATCTAGCTCCATCG	
HapB_qF	GATGCGATGGAACTGTTT	*HapB*
HapB_qR	CCTCCAATGACCCAGCAA	
HapC_qF	CGATACCTGGATAGGTAGA	*HapC*
HapC_qR	TGGCGAGGACATTCTGTTT	
HapE_qF	TCTCCTCTGCATGAATCC	*HapE*
HapE_qR	GACCCAGAAGTAAAGATGA	
Atf1_qF	CGCAAGATGACCGATGAGGA	*Atf1*
Atf1_qR	GGCACTTGAGAGCAGCGA	
NPS6_qF	TCGCAACTATTATCCGTGGAG	*NPS6*
NPS6_qR	CTGGATGGCCCATATACAATG	
MirB_qF	CCTCTCTCCCTACGTCACCA	*MirB*
MirB_qR	ACGGTAGTCAAAGCGGTCAG	
Sit1_qF	CGGTATGATTTGCTGCCACA	*Sit1*
Sit1_qR	ACAGGCATCTACCTCGCTAC	
